# Supplementary material for: Pyruvate Dehydrogenase Kinase 1 inhibition mediated oxidative phosphorylation enhancement in cartilage promotes osteoarthritis progression
Source: BMC Musculoskelet Disord. 2023 Jul 20;24:597. doi: 10.1186/s12891-023-06585-6 (PMC10357736; doi:10.1186/s12891-023-06585-6)
Supplement: Supplementary file 1 — Additional file 1. [file 12891_2023_6585_MOESM1_ESM.zip › MF.Gsea.1653627316242/GOMF_ACETYLGALACTOSAMINYLTRANSFERASE_ACTIVITY.html]

Details for gene set GOMF\_ACETYLGALACTOSAMINYLTRANSFERASE\_ACTIVITY[GSEA]

|  || Dataset | 55457\_collapsed\_to\_symbols.cls.cls #OA\_versus\_normal.cls.cls #OA\_versus\_normal\_repos |
| Phenotype | cls.cls#OA\_versus\_normal\_repos |
| Upregulated in class | OA |
| GeneSet | GOMF\_ACETYLGALACTOSAMINYLTRANSFERASE\_ACTIVITY |
| Enrichment Score (ES) | 0.42153636 |
| Normalized Enrichment Score (NES) | 1.3936665 |
| Nominal p-value | 0.0721393 |
| FDR q-value | 0.3337147 |
| FWER p-Value | 1.0 |
Table: GSEA Results Summary

  

Fig 1: Enrichment plot: GOMF\_ACETYLGALACTOSAMINYLTRANSFERASE\_ACTIVITY      
 Profile of the Running ES Score & Positions of GeneSet Members on the Rank Ordered List

  

| SYMBOL | TITLE | RANK IN GENE LIST | RANK METRIC SCORE | RUNNING ES | CORE ENRICHMENT || 1 | B3GALNT1 | "beta-1,3-N-acetylgalactosaminyltransferase 1 (globoside blood group) [Source:HGNC Symbol;Acc:HGNC:918]" | 101 | 0.914 | 0.1311 | Yes |
| 2 | GALNT11 | polypeptide N-acetylgalactosaminyltransferase 11 [Source:HGNC Symbol;Acc:HGNC:19875] | 164 | 0.845 | 0.2548 | Yes |
| 3 | EXTL2 | exostosin like glycosyltransferase 2 [Source:HGNC Symbol;Acc:HGNC:3516] | 357 | 0.722 | 0.3496 | Yes |
| 4 | GALNT10 | polypeptide N-acetylgalactosaminyltransferase 10 [Source:HGNC Symbol;Acc:HGNC:19873] | 1173 | 0.481 | 0.3595 | Yes |
| 5 | ABO | "ABO, alpha 1-3-N-acetylgalactosaminyltransferase and alpha 1-3-galactosyltransferase [Source:HGNC Symbol;Acc:HGNC:79]" | 1495 | 0.429 | 0.3999 | Yes |
| 6 | CHPF2 | chondroitin polymerizing factor 2 [Source:HGNC Symbol;Acc:HGNC:29270] | 2281 | 0.329 | 0.3890 | Yes |
| 7 | CSGALNACT1 | chondroitin sulfate N-acetylgalactosaminyltransferase 1 [Source:HGNC Symbol;Acc:HGNC:24290] | 2507 | 0.302 | 0.4174 | Yes |
| 8 | GALNT7 | polypeptide N-acetylgalactosaminyltransferase 7 [Source:HGNC Symbol;Acc:HGNC:4129] | 2954 | 0.255 | 0.4215 | Yes |
| 9 | GALNT4 | polypeptide N-acetylgalactosaminyltransferase 4 [Source:HGNC Symbol;Acc:HGNC:4126] | 5610 | 0.023 | 0.2189 | No |
| 10 | GALNT6 | polypeptide N-acetylgalactosaminyltransferase 6 [Source:HGNC Symbol;Acc:HGNC:4128] | 5915 | 0.001 | 0.1955 | No |
| 11 | GALNT1 | polypeptide N-acetylgalactosaminyltransferase 1 [Source:HGNC Symbol;Acc:HGNC:4123] | 6217 | -0.022 | 0.1755 | No |
| 12 | GALNT14 | polypeptide N-acetylgalactosaminyltransferase 14 [Source:HGNC Symbol;Acc:HGNC:22946] | 6762 | -0.060 | 0.1424 | No |
| 13 | GALNT2 | polypeptide N-acetylgalactosaminyltransferase 2 [Source:HGNC Symbol;Acc:HGNC:4124] | 7453 | -0.115 | 0.1063 | No |
| 14 | B4GALNT1 | "beta-1,4-N-acetyl-galactosaminyltransferase 1 [Source:HGNC Symbol;Acc:HGNC:4117]" | 7799 | -0.141 | 0.1010 | No |
| 15 | CHPF | chondroitin polymerizing factor [Source:HGNC Symbol;Acc:HGNC:24291] | 7960 | -0.152 | 0.1116 | No |
| 16 | GALNT8 | polypeptide N-acetylgalactosaminyltransferase 8 [Source:HGNC Symbol;Acc:HGNC:4130] | 8124 | -0.164 | 0.1240 | No |
| 17 | CSGALNACT2 | chondroitin sulfate N-acetylgalactosaminyltransferase 2 [Source:HGNC Symbol;Acc:HGNC:24292] | 9773 | -0.298 | 0.0414 | No |
| 18 | GALNT12 | polypeptide N-acetylgalactosaminyltransferase 12 [Source:HGNC Symbol;Acc:HGNC:19877] | 10414 | -0.362 | 0.0467 | No |
| 19 | GALNT3 | polypeptide N-acetylgalactosaminyltransferase 3 [Source:HGNC Symbol;Acc:HGNC:4125] | 11062 | -0.440 | 0.0634 | No |
| 20 | CHSY1 | chondroitin sulfate synthase 1 [Source:HGNC Symbol;Acc:HGNC:17198] | 11628 | -0.521 | 0.0988 | No |
Table: GSEA details [plain text format]

  

Fig 2: GOMF\_ACETYLGALACTOSAMINYLTRANSFERASE\_ACTIVITY      
 Blue-Pink O' Gram in the Space of the Analyzed GeneSet

  

Fig 3: GOMF\_ACETYLGALACTOSAMINYLTRANSFERASE\_ACTIVITY: Random ES distribution      
 Gene set null distribution of ES for **GOMF\_ACETYLGALACTOSAMINYLTRANSFERASE\_ACTIVITY**
